# A study of the etiology of transient congenital hypothyroidism in Niigata Prefecture, Japan

**DOI:** 10.1186/1687-9856-2013-S1-O55

**Published:** 2013-10-03

**Authors:** Keisuke Nagasaki, Satoshi Narumi, Kiyomi Abe, Tadashi Asami, Hidetoshi Sato, Yohei Ogawa, Toru Kikuchi, Tomonobu Hasegawa, Akihiko Saitoh

**Affiliations:** 1 Division of Pediatrics, Department of Homeostatic Regulation and Development, Niigata University Graduate School of Medical andDental Sciences, Niigata, Japan; 2 Department of Pediatrics, Keio University School of Medicine, Tokyo, Japan; 3 Department of Nursing, Faculty of Nursing, Social Welfare, and Psychology, Niigata Seiryo University, Niigata, Japan

## Background

Several conditions have been known to causetransient congenital hypothyroidism (TCH), including transplacental passage of TSH receptor blocking antibody (TSBAb), maternal antithyroid drug usage, iodine deficiency, iodine excess, fetal prematurity and inactivating*DUOX2*mutations. However, the underlying etiology of TCH is not determined in some cases. In this study, we conducted the first systematic investigation on the etiology of TCH, using screening population-based cohort in Niigata Prefecture, Japan.

## Methods

Between April 2003 and March 2009, 148,100 newborns were screened for CH in Niigata prefecture, and 159 patients were considered positive for CH. We diagnosed patients as having TCH that fulfilled the following two criteria: 1) serum TSH level >30 mU/L and serum FT4 level <1.5 ng/dLat the initial examination, 2) serum TSH level <5 mU/L while investigative discontinuation of thyroxine replacement at 2 years of age. A total of 9 patients (1/16,500) diagnosed with TCH were evaluated. To determine the etiology of TCH, we examined the following: 1) maternal medical history, 2)gestational age and birth weight, 3) maternal anti-thyroid antibodies, 4) urinary iodine concentration at initial visit, and 5) DNA sequence of *DUOX2*.

## Results

Among the nine TCH patients, one had extremely high maternal TSBAb level, one was exposed to propylthiouracil, and two were exposed to excessive iodine.Furthermore, we found that five had biallelic*DUOX2* mutations.

## Conclusions

*DUOX2* mutations were the majorcause of TCH in our cohort study.

